# Sulforaphane promotes ER stress, autophagy, and cell death: implications for cataract surgery

**DOI:** 10.1007/s00109-016-1502-4

**Published:** 2017-01-12

**Authors:** Hanruo Liu, Andrew JO Smith, Simon SR Ball, Yongping Bao, Richard P Bowater, Ningli Wang, I. Michael Wormstone

**Affiliations:** 1 Beijing Institute of Ophthalmology, Beijing Tongren Hospital, Capital Medical University, Beijing, China; 2 School of Biological Sciences, Norwich Research Park, University of East Anglia, Norwich, NR4 7TJ UK; 3 Norwich Medical School, University of East Anglia, Norwich, NR4 7TJ UK

**Keywords:** Sulforaphane, ER stress, Autophagy flux, Lens, Posterior capsule opacification

## Abstract

**Abstract:**

Posterior capsule opacification (PCO) commonly develops following cataract surgery and is a wound-healing response that can ultimately lead to secondary visual loss. Improved management of this problem is required. The isothiocyanate, sulforaphane (SFN), is reported to exert cytoprotective and cytotoxic actions, and the latter may be exploited to treat/prevent PCO. SFN concentrations of 10 μM and above significantly impaired wound-healing in a human lens capsular bag model. A similar pattern of response was also seen with a human lens cell line, FHL124. SFN treatment promoted increased expression of endoplasmic reticulum (ER) stress genes, which also corresponded with protein expression. Evidence of autophagy was observed in response to SFN as determined by increased microtubule-associated protein 1A/1B-light chain 3 (LC3)-II levels and detection of autophagic vesicles. This response was disrupted by established autophagy inhibitors chloroquine and 3-MA. SFN was found to promote MAPK signaling, and inhibition of ERK activation using U0126 prevented SFN-induced LC3-II elevation and vesicle formation. SFN also significantly increased levels of reactive oxygen species. Taken together, our findings suggest that SFN is capable of reducing lens cell growth and viability and thus could serve as a putative therapeutic agent for PCO.

**Key message:**

- SFN reduces lens epithelial cell growth, migration, and viability.
- SFN can promote ER stress and autophagy in lens cells.
- SFN promotes MAPK signaling, and inhibition of MEK can suppress SFN-induced autophagy.
- ER stress and autophagy in lens cells are likely promoted by ROS production.
- SFN may help prevent posterior capsule opacification after cataract surgery.

## Introduction

Cataract renders millions of people blind throughout the world. Despite recent advances in putative cataract treatments, the only currently accepted means of resolving the problem is through surgical intervention [1, 2]. Cataract removal is the most common surgical procedure in the world and is a huge drain on healthcare providers [3, 4]. Posterior capsule opacification (PCO) is the most common complication of cataract surgery and likely the most common cause of nonrefractive decreased postoperative vision [5, 6]. PCO occurs in a significant proportion of cataract surgery patients within 5 years postoperatively, depending on age, geographic location, and the type of intraocular lens (IOL) placed during cataract surgery [2, 7]. PCO reflects the wound-healing process of the lens epithelial cells (LECs) that remain in the capsular bag after cataract surgery. Residual LECs within the capsular bag rapidly grow and proliferate across the posterior lens capsule, which can encroach upon the visual axis and cause a secondary reduction in vision quality [8]. Treatment of PCO is usually straightforward and effective, using the neodymium:YAG (Nd:YAG) laser to cut an opening in the posterior lens capsule, thus clearing the visual axis and restoring vision [2]. However, disruption of the posterior capsule due to complications of cataract surgery results in a relative increase in the occurrence of complications such as an elevation in intraocular pressure, retinal cystoid macular edema, glaucoma, intraocular lens damage, iritis, endophthalmitis, and retinal detachment [9]. Nd:YAG treatment is the second most common corrective surgery, which provides further cost to healthcare providers not to mention the reduced quality of life experienced by the patient as PCO develops. Thus, PCO is an important problem, and thus, improved management of this condition is required. A number of strategies have been proposed but are yet to reach the clinic. These include mechanical approaches, which strive to remove all LECs during surgery or by altering shape and materials of the IOL designs [10, 11]. Different pharmaceutical methods to prevent PCO by removing or destroying residual LECs have also been proposed that can either arrest growth, prevent matrix contraction, or destroy the entire lens cell population [12–15].

In the present study, we investigated sulforaphane [1-isothiocyanato-4-(methylsulfinyl)-butane, sulforaphane (SFN)] in the prevention of PCO. SFN is an organic isothiocyanate that is derived from glucosinolates found in cruciferous vegetables [16]. SFN is an intriguing molecule as it is reported to play a role in both cytoprotection and cytotoxicity. These contrasting outcomes are governed by concentration, such that a threshold is eventually reached that is associated with reduced cell viability and death. In the lens, we have previously shown that low micromolar concentrations of SFN do not reduce cell viability or promote cell death [17]. At these lower concentrations, Nrf2 signaling is activated, which leads to increased expression of antioxidant response proteins and thus better prepares cells to manage oxidative stress. Similar responses have also been observed in other cells and tissue [16]. SFN was also found to prevent oxidative stress-induced opacity of cultured whole lenses [17]. With respect to cytotoxic actions of SFN, it has been reported to inhibit tumor growth in many in vivo models by inducing cell cycle arrest and instigating apoptosis [18, 19]. Also, SFN has been proposed to reduce proliferation and promote cell death via ROS generation [20, 21].

In the present study, we aimed to assess whether SFN could reduce cell growth and promote cell death using a human tissue culture model and a human lens cell line, using them as a tool to identify mechanisms that drive these different cellular outcomes. In particular, we elected to investigate the potential involvement of endoplasmic reticulum (ER) stress pathways [22, 23] and autophagy [24, 25]. ER stress is reported to be enhanced by SFN [26, 27]. Similarly, SFN can also initiate autophagy in several cell types [28], which in addition to its housekeeping role can also contribute to cell death in certain circumstances [29]. Our investigation revealed a cytotoxic action of SFN in our human lens capsular bag model, which is an excellent predictive tool for clinical outcomes. Using a human lens cell line, we also identified that SFN can promote ER stress and autophagy in human lens cells. In the case of autophagy, SFN-mediated MAPK signaling appears to play an important role.

## Materials and methods

### Human lens capsular bag preparation

Simulated cataract operations were performed to create capsular bags from human donor lenses [30] that were obtained with informed consent and used in accordance with the tenets of the Declaration of Helsinki. Approval for the study and experimental protocols (04/Q0102/57) was granted by a national research ethics committee under the Health Research Authority (UK). Using an insulin needle, the anterior capsule was breached approximately 3 mm from the equator, and an incision was made from that point to the center of the capsule. By tugging the flap, created by this incision, with surgical forceps, a continuous curvilinear capsulorhexis was created, such that a disc of anterior capsule was removed, leaving an opening approximately 5 mm in diameter. The resultant window enabled the lens fiber mass to be removed by hydroexpression. Residual fibers were removed by joint irrigation with Hartmann’s solution and aspiration. The resultant capsular bag was then dissected free of the zonules and secured on a sterile 35-mm polymethylmethacrylate (PMMA) Petri dish. Eight entomological pins (Anglian Lepidopterist Supplies, Hindolveston, Norfolk, UK) were inserted through the edge of the capsule to retain its circular shape. Capsular bags were maintained in 1.5 mL Eagle’s minimum essential medium (EMEM) (Sigma-Aldrich, Poole, UK) and incubated at 35 °C in a 5% CO_2_ atmosphere. Preparations were exposed to 0, 1, 10, or 100 μM SFN for the first 24 h of culture and then maintained in unsupplemented EMEM for the remaining experimental period (end point at 30 days). Ongoing observations were made using a Nikon phase-contrast microscope (Nikon, Tokyo, Japan).

### FHL124 human lens cell line

FHL124 is a nonvirally transformed cell line generated from human capsule-epithelial explants, showing a 99.5% homology (in transcript profile) with the native lens epithelium [31]. FHL124 cells were routinely cultured at 35 °C in a humidified atmosphere of 95% air and 5% CO_2_, in EMEM supplemented with 5% fetal calf serum (FCS) (Gibco, Paisley, UK) and 50 μg/mL gentamicin (Sigma-Aldrich). FHL124 cells were seeded on the following: 35-mm tissue culture dishes (30,000/dish for Western blot, qRT-PCR, TEM, and scratch migration assay), coverslips (10,000) for immunocytochemistry, and 96-well plates (5000/well for MTS (3-(4,5-dimethylthiazol-2-yl)-5-(3-carboxymethoxyphenyl)-2-(4-sulfophenyl)-2H-tetrazolium) assay and ROS detection assay (Promega, Madison, WI) and lactate dehydrogenase (LDH) assay (Roche).

### Cell viability

A cell proliferation assay (CellTiter 96 AQueous; Promega) was used in accordance with the manufacturer’s instructions to assess FHL124 cell viability. This assay is a colorimetric method for determining the number of viable cells in proliferation. The assay is based on the cellular conversion of a tetrazolium salt (MTS) into a formazan product. The resultant absorbance is directly proportional to the number of viable cells in culture. Absorbance was measured at 490 nm with a spectrophotometric plate reader (FLUOstar Omega plate reader; BMG Labtech).

### Cell death assay

A nonradioactive cytotoxicity assay (CytoTox 96R; Roche, Welwyn Garden City, UK) was used to measure the release of LDH from cultured human lens cells. The procedure followed the manufacturer’s protocol. The plate was read at 490 nm with a FLUOstar Omega plate reader (BMG Labtech).

### Scratch wound assay

FHL124 cells in each well were allowed to grow to 95% confluence. Cells were then placed in unsupplemented EMEM for a 24-h period. A scratch was made through the sheet of cells using a plastic pipette tip. Photomicrographs were taken immediately after the scratch was made and after 24 h of incubation in experimental conditions. ImageJ1.45s analysis software (available in the public domain at http://rsbweb.nih.gov/ij/) was then used to quantify the initial scratch area and the final area of the scratch.

### TaqMan qRT-PCR

qRT-PCR reactions were performed using an ABI prism 7700 Sequence Detection System (Applied Biosystems, Warrington, UK) under the following conditions: 50 °C for 2 min, 95 °C for 10 min, and then 40 cycles, each consisting of 15 s at 95 °C and 1 min at 60 °C. Each reaction was performed in 25 μL and contained reverse transcribed RNA, primers, and probes (sequences for primers and probes are given in Table 1) and TaqMan PCR master mix (Applied Biosystems). Primers and probes were bought as predesigned TaqMan probe and primer sets provided by Applied Biosystems. The threshold cycle (Ct) values, defined as the point at which the fluorescent signal is recorded as statistically above background, were obtained using the 7500 Fast system software 2.0.5 (Applied Biosystems).

**Table 1:** Predesigned TaqMan probe/primer sets for genes of interest

Gene name	Protein encoded	Ref seq	TaqMan primer/probe set
AFT6	AFT6	NM_007348	Hs00232586_m1
ERN1	IRE1	NM_001433	Hs00176385_m1
EIF2AK3	EIF2α	NM_003836.3	Hs00178128_m1
HSPA5	BiP	NM_004836.3	Hs99999174_m1

### Immunoblotting

Cell lysates from FHL124 cells were prepared using Daub’s lysis buffer supplemented with 1 mM phenylmethylsulfonyl fluoride (PMSF) and 10 μg/mL aprotinin for 20 min on ice and centrifuged at 16,060×*g* for 10 min. The protein content was determined by the BCA assay (Bio-Rad, Hemel Hempstead, UK) so that equal amounts of protein per sample were loaded onto 8% SDS–polyacrylamide gels and transferred to PVDF membrane using a semidry transfer cell. The membrane was blocked with PBS containing 5% nonfat dry milk and 0.1% Tween-20, hybridized with primary antibody (anti-LC3, (Sigma-Aldrich, Poole, Dorset); anti-ERK, anti-JNK, anti-p38, anti-β-actin (Cell Signaling Technology, Beverly, MA, USA), anti-EIF-2α, anti-BiP/GRP78 (BioSource International, Rockville, MD); anti-IRE1, anti-ATF6 (Abcam, Cambridge, UK)) followed by incubation with secondary antibody (Amersham Biosciences, Bucks, UK). Proteins were detected using the ECL plus blotting analysis system (Amersham Biosciences).

### Transmission electron microscopy

Cultured FHL124 were treated with 100 μM SFN for 24 h (*n* = 4) to fixation with 3% gluteraldehyde in 0.1 M phosphate buffer (pH 7.2) and 2% paraformaldehyde (PFA) for 24 h. Following fixation, samples were post-fixed in 1% OsO_4_ in the same buffer for 30 min. Appropriate areas for thin sectioning were cut at 70 nm and stained with saturated 2% uranyl acetate and 2% lead citrate before examination on a transmission electron microscope (Libra 120, Carl Zeiss) at 120 kV.

### Immunofluorescence

Cells were maintained in unsupplemented EMEM for 24 h before being placed in experimental conditions for selected periods. Cells were fixed with 4% formaldehyde in PBS for 30 min and permeabilized with PBS containing 0.5% Triton X-100 for 30 min. Preparations were washed three times for 5 min in PBS containing 0.02% *w*/*v* BSA and 0.05% *v*/*v* IGEPAL. Nonspecific sites were blocked with normal goat or donkey serum (1:50 in 1% *w*/*v* BSA in PBS). Following removal of the blocking buffer, rabbit polyclonal primary antibody against microtubule-associated protein 1A/1B-light chain 3 (LC3) (Sigma-Aldrich, Poole, Dorset) diluted 1:200 was applied overnight at 4 °C. Cells were subsequently washed with PBS and placed in ALEXA-488 conjugated secondary antibody (1:250; Invitrogen) for 1 h at room temperature. The stained preparations were again washed extensively and mounted on microscope slides with Hydromount mounting medium (National Diagnostics, Hull, UK). Images were viewed using fluorescence microscopy (Axioplan 2; Zeiss), and applicable images were quantified using ImageJ1.45s analysis software (available in the public domain at http://rsbweb.nih.gov/ij/).

### ROS detection assay

ROS levels were measured using a cellular reactive oxygen species detection assay (Abcam, UK) that uses the cell permeant reagent 2′,7′-dichlorofluorescin diacetate (DCFDA) to measure hydroxyl, peroxyl, and other ROS activities. This was in accordance with manufacturer instructions. The cells were washed with buffer before being stained with 20 μM DCFDA for 45 min at 37 °C, and then washed with buffer again before addition of EMEM and test compounds. The fluorescence (excitation/emission was 485/535 nm, respectively) was then measured following a 2-h incubation.

### Statistical analysis

A Student’s *t* test analysis was performed using Excel software (Microsoft, Redmond, WA) to determine any statistical difference between two groups. One-way ANOVA with Tukey’s post hoc analysis was used to assess multiple groups when all or many pairwise comparisons were of interest. One-way ANOVA with Dunnett’s post hoc analysis was used to assess all groups compared against the control group. A 95% confidence interval was used to assess significance.

## Results

### SFN can reduce lens cell coverage and promote cell death

**Fig. 1:**
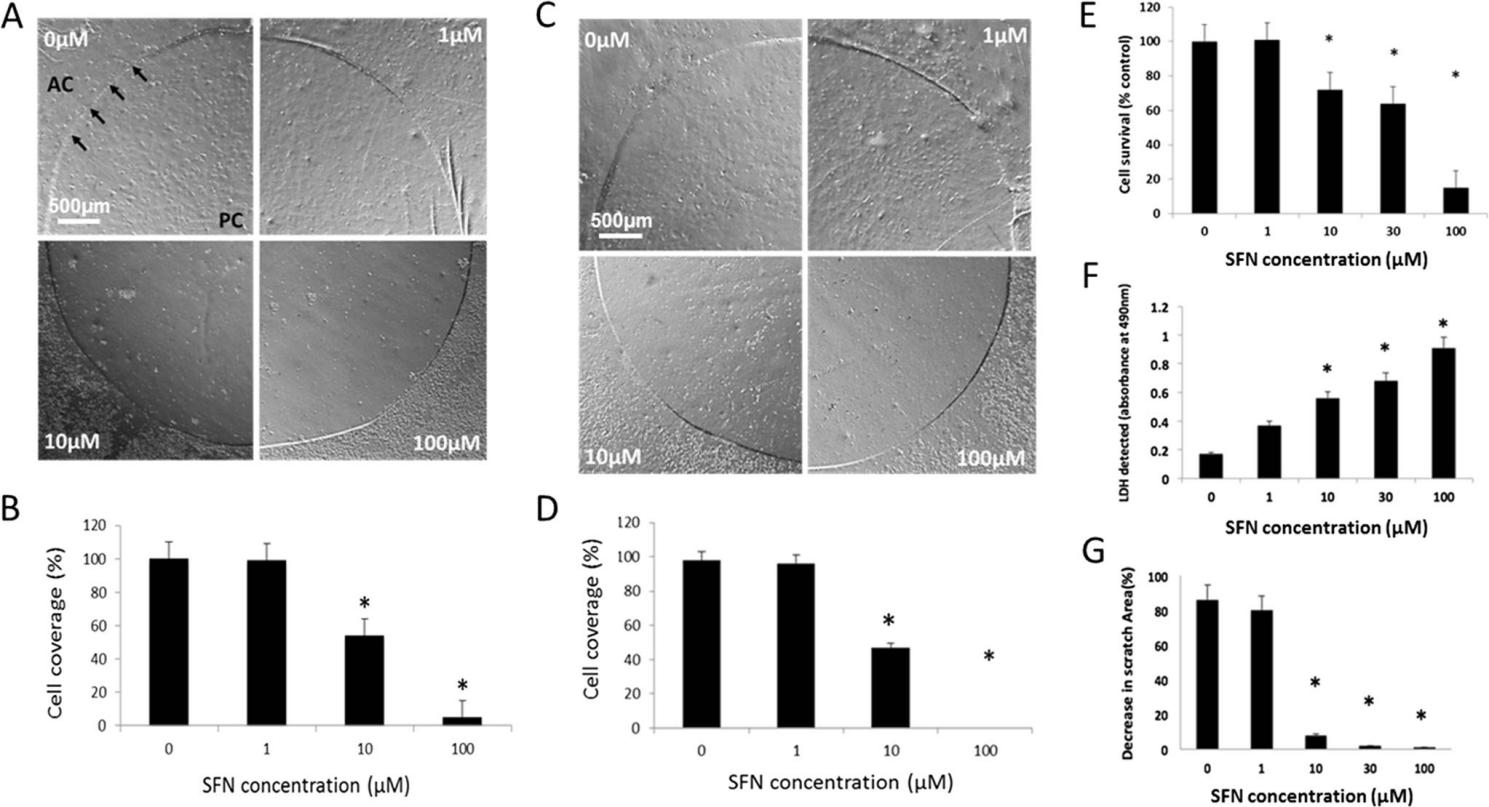
The effect of SFN on cell survival and growth of human lens cells. Modified dark-field images of four human capsular bag quarters showing the posterior capsule (*PC*), capsulorhexis (*arrowed*), and outer anterior capsule (*AC*) captured after **a** 8 and **c** 30 days. Cell coverage on the posterior capsule was quantified from dark-field images at **b** 8 and **d** 30 days. Data are presented as mean ± SEM (*n* = 4). *Asterisk* indicates a significant difference between treated and control groups. The effects of SFN exposure to FHL124 cells over a 24-h period on **e** cell viability, **f** cell death, and **g** migration assessed using the MTS, LDH, and scratch wound assays, respectively. The data are presented as mean ± SEM of four independent experiments. *Asterisk* represents a significant difference between untreated control and treatment groups (*p* ≤ 0.05; ANOVA with Dunnett’s post hoc test)

Capsular bags maintained in standard serum-free culture conditions demonstrated progressive cell growth across denuded regions of the anterior capsule, the outer anterior capsule, and, importantly, the previously cell-free posterior capsule. At day 8, cells could be clearly seen on the central posterior capsule. The level of growth in capsular bag preparations treated with 1 μM SFN for the first 24-h period of culture was similar to control preparations (Fig. 1a, b). Cells were also observed on the central posterior capsule with 10 μM SFN, but growth was retarded (Fig. 1a, b). Limited coverage of the central posterior capsule was seen with 100 μM SFN, and indeed, the cells on the anterior capsule appeared distressed at this time point (Fig. 1a, b). Following 30 days of culture (end point), control capsular bags exhibited complete coverage of the posterior capsule (Fig. 1c, d). Exposure to 1 μM SFN for the first 24 h of culture had negligible effect on cell coverage by day 30 (Fig. 1c, d). Capsular bags exposed to 10 μM SFN for 24 h demonstrated a marked reduction in cells growing on the posterior capsule, and 24-h treatment with 100 μM SFN leads to widespread cell death and completely inhibited coverage of the posterior capsule (Fig. 1c, d).

Previous work has shown that SFN can reduce cell viability and induce death (by apoptosis) of FHL124 cells [17]; this was determined using the ApoTox-Glo Triplex assay (Promega). In the current study, we verified this response using different biochemical assays. The MTS assay was used to assess cell viability, and it was found that SFN reduced cell viability in a concentration-dependent manner, such that significant differences from control were seen with treatments at 10 μM and above (Fig. 1e). Cell damage/death was observed using the LDH assay, and in this case, significant increases in LDH levels were detected with 10-, 30-, and 100-μM treatments (Fig.1f).

We further assessed the impact of SFN on wound-healing of FHL124 cells using the scratch assay method. Following wound formation, control and 1 μM SFN-treated cells were seen to close the denuded region at similar rates. However, significant inhibition of wound closure was observed with 10-, 30-, and 100-μM SFN treatments (Fig. 1g).

The findings with the cell line demonstrate that this system is a relevant model to study SFN in relation to PCO and lens cell behavior. We therefore employed this biological tool to further investigate the mechanisms governed by SFN that could contribute to abrogation in the wound-healing response.

### Promotion of ER stress pathways by SFN

**Fig. 2:**
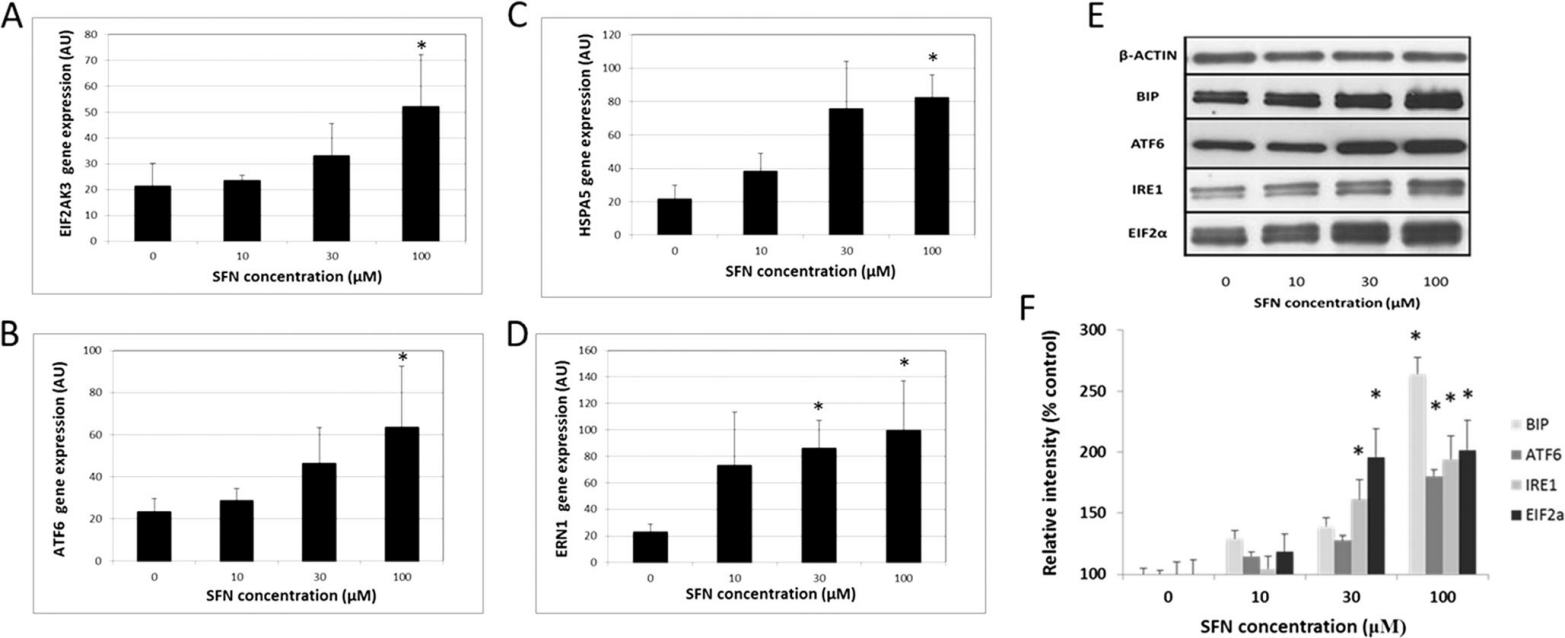
SFN can induce ER stress in FHL124 human lens epithelial cells. Gene expression of **a** EIF2AK3, **b** ATF6, **c** HSPA5, and **d** ERN1 following 24-h exposure to SFN concentrations, detected by real-time PCR. Values were normalized to 18S gene expression. The data represent mean ± SEM (*n* = 4). *Asterisk* represents a significant difference between untreated control and treated groups (*p* ≤ 0.05; ANOVA with Dunnett’s post hoc test). Detection of ER stress proteins in response to SFN was determined using Western blot methods. **e** Representative blots showing BiP, ATF6, IRE1, EIF2α, and β-actin levels within FHL124 cells; product bands were observed at ∼78, 75, 107, 36, and 45 kDa, respectively. **f** Quantitative data derived from band intensities; the protein band intensities for BiP, ATF6, IRE1, and EIF2α were normalized to β-actin. Data are presented as mean ± SEM (*n* = 4). *Asterisk* indicates a significant difference between treated and nontreated control groups (*p* ≤ 0.05; ANOVA with Dunnett’s post hoc test)

In order to investigate the putative involvement of ER stress in SFN-mediated events, real-time PCR and Western blot analysis were employed. FHL124 cells were treated with 10, 30, and 100 μM SFN for 24 h. Real-time PCR revealed increases in all four ER stress gene products tested in FHL124 cells after exposure to SFN for 24 h (Fig. 2). The level of the inositol-requiring enzyme 1 (IRE1) gene (ERN1) in lens cells was significantly increased within 24 h of exposure to 30 μM SFN (Fig. 2d). All genes were significantly elevated at 100 μM SFN (Fig. 2a–d). At the protein level, binding immunoglobulin protein (BiP), activating transcription factor 6 (ATF6), eukaryotic translation initiation factor 2α (EIF2α), and IRE1 were significantly upregulated in response to a 24-h 100-μM SFN treatment. When cells were exposed to 30 μM SFN, there were significant increases in IRE1 and EIF2α expression (Fig. 2e, f).

### SFN can promote autophagy in lens cells

**Fig. 3:**
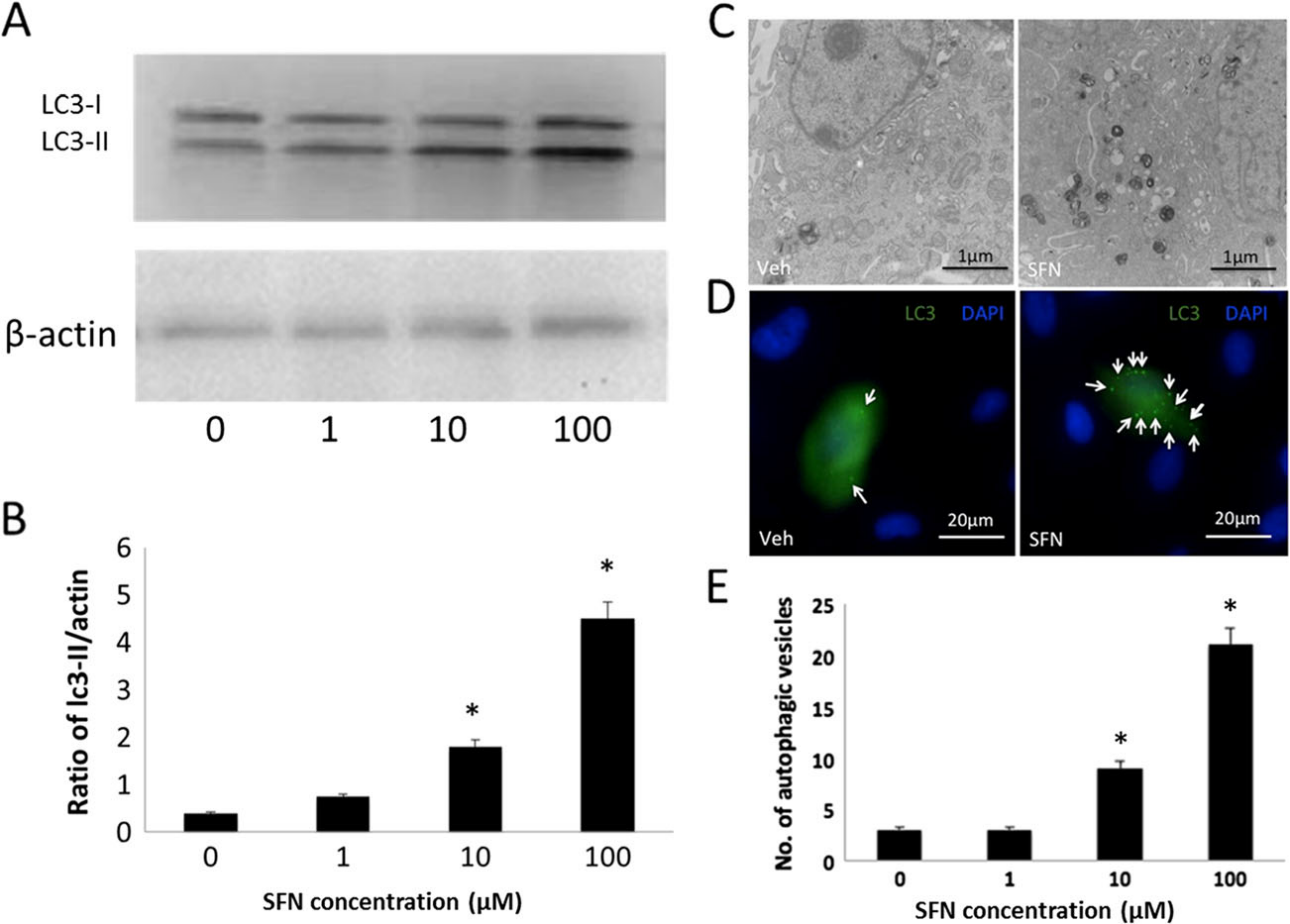
SFN can initiate autophagy in FHL124 human lens epithelial cells. LC3-I and LC3-II levels were detected using Western blot methods following 24-h exposure to increasing concentrations of SFN (**a**, **b**); a representative blot showing products for LC3-I and LC3-II at 18 and 16 kDa (**a**) and quantitative data (**b**) are displayed. Data are presented as mean ± SEM (*n* = 4). *Asterisk* indicates a significant difference between treated and nontreated control groups (*p* ≤ 0.05; ANOVA with Dunnett’s post hoc test). Transmission electron micrographs demonstrate the ultrastructure of cells treated with 100 μM SFN (**b**). A number of autophagic vesicles can be clearly seen with SFN treatment. Fluorescence micrographs showing LC3 distribution in association with autophagic vesicles in control and 100 μM SFN-treated cells (**c**, **d**); representative images (**c**) and quantitative data pooled from three separate experiments (**d**) are presented. Data are presented as mean ± SEM. *Asterisk* indicates a significant difference between the treated group and untreated controls (*p* ≤ 0.05; ANOVA with Dunnett’s post hoc test)

To examine whether SFN could induce autophagy in LEC cells, FHL124 cells were treated with 1, 10, and 100 μM SFN for 24 h. The level of LC3, a well-known protein associated with autophagosome membranes, was analyzed by Western blot. While expression of LC3-I was consistent in all treatment groups, the level of LC3-II in cells treated with SFN was significantly increased compared to control cells without SFN treatment (Fig. 3a, b). To provide further evidence of autophagy in response to SFN, we examined cells using transmission electron microscopy (Fig. 3c) and immunofluorescence (Fig. 3d, e). Using these methods, we could observe increased numbers of autophagosomes in cells treated with 10 and 100 μM SFN.

**Fig. 4:**
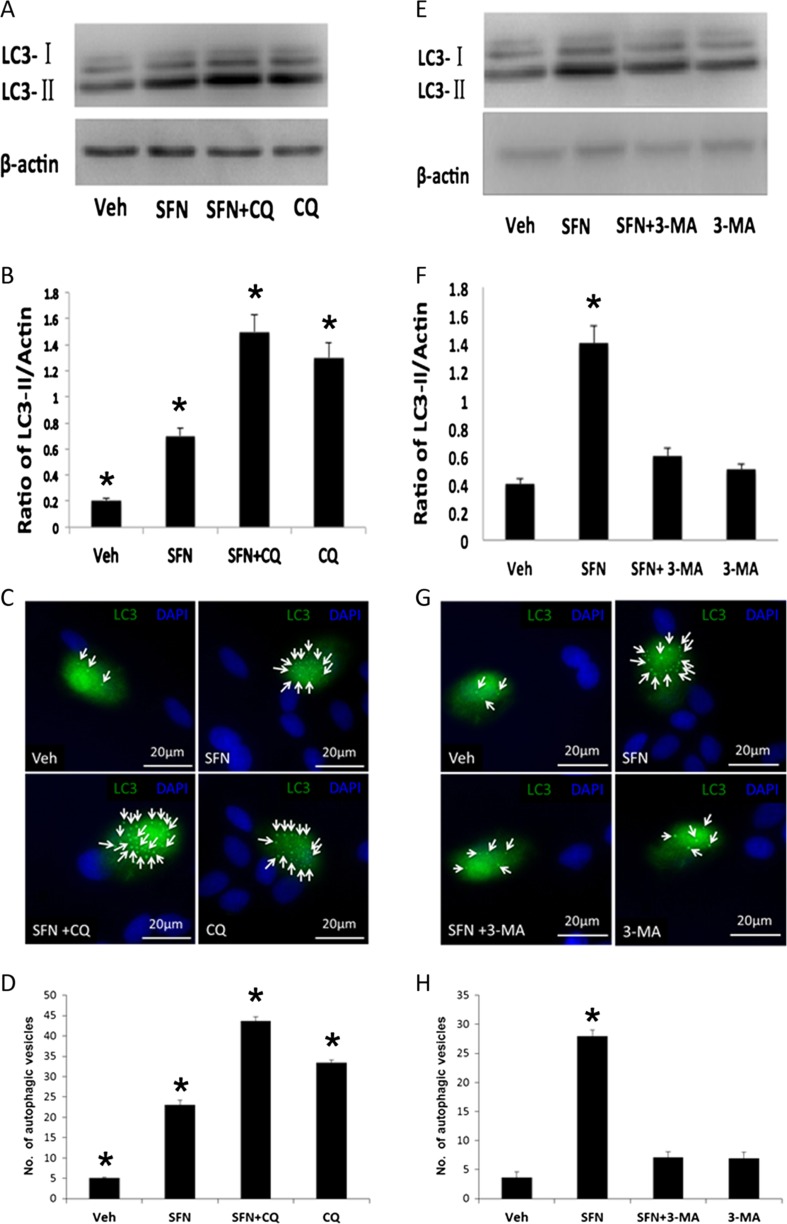
Chloroquine and 3-MA disrupt SFN-induced autophagy responses in FHL124 human lens epithelial cells. LC3 levels detected using Western blot methods and autophagic vesicle formation in response to 100 μM SFN treatment in the presence and absence of 50 μM chloroquine (**a**–**d**) and 500 μM 3-MA (**e**–**h**). Western blots detected products for LC3-I and LC3-II at ∼18 and 16 kDa; a band corresponding to β-actin was detected at 45 kDa. Data are presented as mean ± SEM (*n* = 3). *Asterisk* indicates a significant difference from all other groups (*p* ≤ 0.05; ANOVA with Tukey’s post hoc test)

The increase of LC3-II could result from several events, including an interruption in autophagosome–lysosome fusion, inhibiting lysosome-mediated proteolysis or raising the lysosomal pH [29]. To further clarify whether the increased level of LC3-II by SFN treatment was caused by interrupting the autophagosome–lysosome fusion or not, we treated FHL124 cells with 100 μM SFN in the presence of 50 μM chloroquine (CQ) which is a classic lysosomal inhibitor, for 6 h (Fig. 4a–d). Using this approach, we observed a significant increase in LC3-II levels relative to control when SFN and CQ were added alone. Application of SFN and CQ together led to a further significant increase. These results were also mirrored using immunofluorescence, which demonstrated significant changes in autophagic vesicle numbers with SFN and CQ, which were further elevated with co-treatment. To further assess the hypothesis that SFN can induce autophagy in FHL124 cells, 3-methyladenine (3-MA) was utilized as an early-stage autophagy inhibitor. Treatment with 100 μM SFN induced significant increases in LC3-II levels and autophagosome numbers. These responses were significantly suppressed by co-treatment with 500 μM 3-MA (Fig. 4e–h).

### MAPK signaling is critical for SFN-induced autophagy

**Fig. 5:**
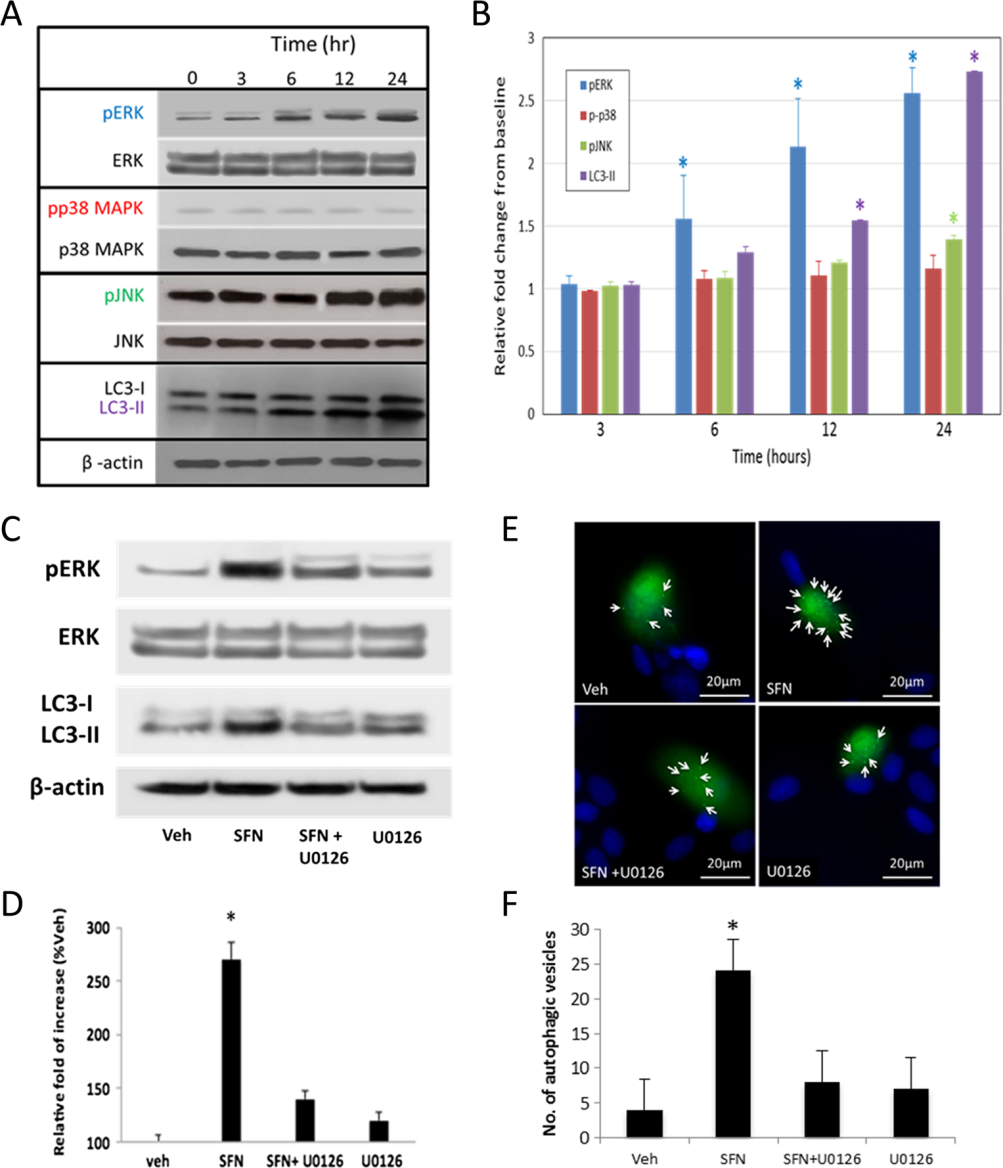
MAPK signaling involvement in SFN-induced autophagy in FHL124 human lens epithelial cells. The effects of 100 μM SFN on p38, JNK, and ERK phosphorylation in conjunction with LC3-II levels detected by Western blot methods (**a**, **b**); representative blots showing products at ∼44/42 kDa (phospho and total ERK1/2), 46 kDa (phospho and total JNK1), 38 kDa (phospho and total p38), and 45 kDa (β-actin) (**a**) and pooled quantitative data (**b**) are presented. Data are presented as mean ± SEM (*n* = 4). *Asterisk* indicates a significant difference between treated and nontreated control groups (*p* ≤ 0.05; ANOVA with Dunnett’s post hoc test). The influence of inhibiting ERK phosphorylation (using 5 μM U0126) on SFN induced LC3-II expression (**c**, **d**) and autophagic vesicles (**e**, **f**). Western blots detected products for LC3-I and LC3-II at ∼18 and 16 kDa; phospho and total ERK1/2 were detected at ∼44/42 kDa; a band corresponding to β-actin was detected at 45 kDa. Data are presented as mean ± SEM (*n* = 3). *Asterisk* indicates a significant difference from all other groups (*p* ≤ 0.05; ANOVA with Tukey’s post hoc test)

**Fig. 6:**
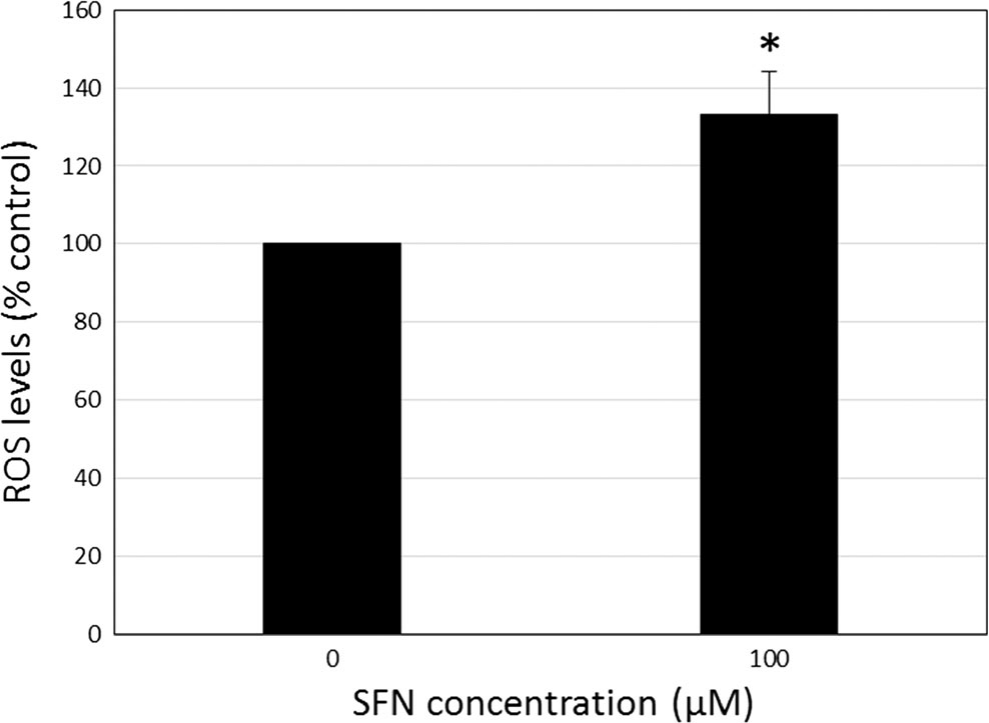
SFN can increase ROS production in FHL124 human lens epithelial cells. The effect of 100 μM SFN on ROS production was detected 2 h following initial exposure. Data are presented as mean ± SEM (*n* = 4). *Asterisk* indicates a significant difference between treated and nontreated control groups (*p* ≤ 0.05; Student’s *t* test)

The mitogen-activated protein kinases (MAPKs) have been reported to promote autophagy as downstream mediators of ROS [28]. Therefore, we analyzed the level of several phosphorylated MAPKs including p38, c-Jun N-terminal kinase (JNK), and ERK by immunoblotting to examine if MAPKs are involved in SFN-induced autophagy in FHL124 cells. One hundred micromolars SFN did not induce a detectable change in the level of phospho-specific p38 at any time point tested. A significant but relatively weak increase in pJNK level was observed at the 24-h time point. In contrast, levels of phosphorylated ERK1/2 (pERK) in cells treated with SFN (100 μM) showed significant increases at 6-, 12-, and 24-h time points (Fig. 5a, b). This increase in ERK activation preceded detectable changes in LC3-II levels (Fig. 5a, b). These results indicate that ERK activation might be involved in SFN-induced autophagy. We therefore tested this notion by disrupting ERK activation using the MEK inhibitor U0126 (Fig. 5c–f). FHL124 cells were pretreated with U0126 (5 μM) for 30 min before SFN treatment. Application of U0126 significantly reduced SFN-induced increase in LC3-II levels and autophagic vesicle incidence. Finally, it has been suggested that SFN-induced activation of ERK is mediated by ROS. We therefore determined whether SFN could induce ROS levels in FHL124 cells. Application of 100 μM SFN was found to significantly increase ROS relative to controls (Fig. 6).

## Discussion

The influence of SFN on the physiology of human cells has been studied extensively in recent years, but this work has largely focused on cancer [16, 32]. However, the functional role of SFN in the prevention of PCO, which affects a large proportion of cataract patients, has not been investigated. In the present study, it was therefore deemed an important opportunity to test the hypothesis that SFN can inhibit the development of PCO. Evaluation of SFN in a human lens capsular bag model supported this notion, and the effective concentrations required mirrored results obtained in a human lens epithelial cell line, FHL124. This therefore suggested that the readily available cell line could be employed to establish mechanisms that underpin SFN-induced effects.

The present study demonstrates that SFN can induce ER stress, which was observed by induction of ER stress gene and protein expression. An important role of the ER is to sense environmental and physiological stresses. This response is mediated by activation and repression of pathways resulting in specific functional outcomes. The ER is the site of protein synthesis, where folding and trafficking are initiated, and also the mediator of internal and external stresses [22]. ER stress can arise because unfolded or misfolded proteins are produced within the ER. Normal levels of unfolded proteins can be counteracted by a number of ER chaperone proteins, but if the level continues to increase, for example, through prolonged oxidative or osmotic stress, then it is detected by the molecule BiP, which in turn activates one or more of three stress pathway initiators (pERK, IRE1, and ATF6). Once a prolonged stress is sensed, then a range of external pathways are initiated and cell death through apoptosis can result [23].

Application of the classical ER stress molecule thapsigargin leads to an inhibition of lens cell growth and, ultimately, to cell death through apoptosis [33, 34]. It has also been reported that diabetic and oxidative stresses to lens cells can give rise to an ER stress response, seen as an increase in BiP production and caspase activation [35]. Arsenic trioxide (As_2_O_3_) is also known to promote ER stress and can reduce lens cell viability [36]. Combinatorial treatment with SFN and As_2_O_3_ or other known ER stressors such as thapsigargin has been employed in the management of different cancers [26, 27] and therefore might provide a promising therapeutic approach for PCO.

It has been suggested that SFN can increase ROS production [20, 28], and in the current study, we confirm that lens cells show elevated ROS levels in response to SFN. Interestingly, as discussed above, SFN exposure in combination with As_2_O_3_ can result in a dramatic increase in levels of ROS compared to treatment with either agent alone [26]. SFN, alone or with As_2_O_3_, decreased intracellular glutathione (GSH) content. Furthermore, addition of the free radical scavenger *N*-acetyl-l-cysteine (NAC) rescued cells from As_2_O_3_/isothiocyanate-mediated cytotoxicity [26]. As As_2_O_3_ has been associated with ER stress in the lens and investigated for its potential benefits in the prevention of PCO [36], this suggests that SFN deserves further investigation in combination with As_2_O_3_ in the treatment of PCO and it will be interesting to establish the role of ROS in SFN-induced ER stress.

Autophagy is a highly controlled process that can be affected by several conditions such as metabolic stress, ER stress, oxidative stress, and hypoxia [37]. The process of autophagy involves the formation of double-membraned vesicles (autophagosomes), which encapsulate the cytoplasm and organelles and fuse with lysosomes, leading to degradation of the contents of the vesicle. It has been associated with specific pathologies including cancer, liver disease, and neurodegeneration by a growing number of studies [38]. Brennan et al. [39] established that autophagy occurs in normal human lens cells, with 42 autophagy genes expressed and a number of autophagosomal proteins detected in both the lens epithelium and fibers. The housekeeping role of autophagy may be particularly important in normal lens maintenance, homeostasis, and fiber differentiation [40, 41]. The loss of lens-specific autophagy-related 5 (Atg5) has been reported to result in age-related cataract formation, and Pik3c3/Vps34 genes are shown to leading cortical cataract in lens [42]. In the present study, we clearly show that SFN is capable of promoting autophagy flux, which occurs in association with reduced viability and increased cell death.

With respect to PCO, previous work using in vitro canine lens capsular bags has indicated that cyclosporine A (CsA)-induced cell loss is attributed to an autophagy-related mode of cell death rather than classical apoptosis [43]. While autophagy is typically linked to general maintenance of cells, its relationship with cell death is beginning to emerge [29] and further investigation of the links between cell death and autophagy in response to SFN will be of interest in the future.

ERK is a member of the MAPK family that is involved in many aspects of cell biological functions such as proliferation, migration, differentiation, and death [44, 45]. MAPKs such as p38, ERK, and JNK have been found to have downstream effects of ROS in autophagy induction [46, 47]. Here, we showed that SFN can induce ROS and activate ERK, which in turn facilitates autophagy in human lens epithelial cells. It is speculated that SFN generates ROS, which could activate ERK. A link between ROS and ERK activation is recognized, and it is proposed that this event could occur through several putative mechanisms [48]. One possibility is the activation of upstream growth factor receptors, such as EGF receptor, which have been reported to be activated in response to oxidative stress [49]. EGFR is expressed in both FHL124 cells and the native lens. It is also possible that ROS could alter protein structure of signaling molecules and in doing so promote signaling activity [48]. It is also feasible that MAPK phosphatases, the negative regulators of MAPK signaling, are deactivated and degraded by ROS [48]. Removal of this negative regulator would therefore enable persistent activation of ERK to take place and may best explain the results observed. Signaling mediated through ERK is often associated with growth and survival, but it is also linked to death pathways [45]. It has been suggested that ERK activity can play a role in mediating extrinsic and intrinsic apoptotic pathways. In the case of SFN treatment, sustained ERK activity is likely to facilitate promotion of the intrinsic apoptotic pathway that is associated with release of pro-apoptotic factors from the mitochondrion that will lead to caspase-9 activation, which will then activate caspase3/7 [45]. The role of ERK in autophagy is also very interesting. Autophagy itself serves an important role in cell maintenance but in some cases can itself lead to cell death [29, 45]. The mode of cell death could be through conventional apoptosis, but it is also often reported that a distinct autophagy-induced cell death can arise that not only has some features common with conventional apoptosis but also presents distinct morphological changes leading to death. It will be of great interest to tease out the relationship between ROS, ERK, and cell death using free radical scavengers and MEK inhibitors to achieve this.

Considering the data as a whole, it is possible to speculate on the sequence of events that may take place following SFN application that ultimately lead to cell death. The primary initiating event is likely to be the generation of free radicals within the cell that will result in an oxidative stress that triggers an ER stress response and promote ERK activity. These two events are both known to stimulate autophagy, and, thus, increased breakdown of cellular material will result. ER stress is associated with reduced protein synthesis, and, thus, a restricted capacity to replace damaged organelles will compromise the cells and trigger death pathways. Sustained ERK activity is likely to deplete mitochondrial numbers, which will in turn activate caspase-mediated cell death. Similarly, a sustained ER stress could also promote caspase-mediated cell death pathways.

In summary, we have shown that SFN can reduce lens cell viability in both a human lens cell line and a tissue culture model, which has implications for the treatment of PCO. Moreover, we have demonstrated for the first time that SFN can promote ER stress and autophagy in lens cell. SFN-induced autophagy requires MAPK signaling, and we speculate that both ER stress and autophagy are regulated by ROS production.
